# Discipline and punishment in panoptical public goods games

**DOI:** 10.1038/s41598-024-57842-0

**Published:** 2024-04-04

**Authors:** Rocio Botta, Gerardo Blanco, Christian E. Schaerer

**Affiliations:** 1 Polytechnic School, National University of Asuncion, San Lorenzo, Paraguay; 2 Pontificia Universidad Católica de Valparaíso, Valparaíso, Chile

**Keywords:** Computational models, Human behaviour, Applied mathematics

## Abstract

In Public Goods Games (PGG), the temptation to free-ride on others’ contributions poses a significant threat to the sustainability of cooperative societies. Therefore, societies strive to mitigate this through incentive systems, employing rewards and punishments to foster cooperative behavior. Thus, peer punishment, in which cooperators sanction defectors, as well as pool punishment, where a centralized punishment institution executes the punishment, is deeply analyzed in previous works. Although the literature indicates that these methods may enhance cooperation on social dilemmas under particular contexts, there are still open questions, for instance, the structural connection between graduated punishment and the monitoring of public goods games. Our investigation proposes a compulsory PGG framework under Panoptical surveillance. Inspired by Foucault’s theories on disciplinary mechanisms and biopower, we present a novel mathematical model that scrutinizes the balance between the severity and scope of punishment to catalyze cooperative behavior. By integrating perspectives from evolutionary game theory and Foucault’s theories of power and discipline, this research uncovers the theoretical foundations of mathematical frameworks involved in punishment and discipline structures. We show that well-calibrated punishment and discipline schemes, leveraging the panoptical effect for universal oversight, can effectively mitigate the free-rider dilemma, fostering enhanced cooperation. This interdisciplinary approach not only elucidates the dynamics of cooperation in societal constructs but also underscores the importance of integrating diverse methodologies to address the complexities of fostering cooperative evolution.

## Introduction

Cooperation plays a central role in nature’s grand stage. Just as a hive’s functionality hinges on the collective efforts of bees and the forest ecosystem thrives through reciprocal interactions among its species, so do human endeavors profoundly depend on cooperation. Yet, a paradoxical challenge arises against this backdrop of intrinsic cooperative synergy. The temptation of free-riding-reaping the rewards without equivalent contribution-presents a significant obstacle.

Thus, even before its selection in 2005 as one of twenty-five major questions facing science^1^, studying the foundations and barriers of cooperative behavior has mobilized a significant interdisciplinary effort to understand how cooperation spontaneously develops and evolves among living entities, encompassing a broad spectrum of scientific fields.^2–7^

Decades of academic research have shed light on the evolution of cooperation, and several studies have proposed mechanisms to represent its dynamic, including kin-selection^2^, reciprocity^3,8^, structural frameworks^9,10^, and incentives such as punishments and rewards^11^. These mechanisms have undergone thorough analysis through the application of game theory principles^12^, particularly evolutionary game theory^13^, to explore the dynamics of populations^14–16^. In this context, Public Goods Games (PGG) have been extensively employed to explore social dilemmas across populations^17^, examining various settings such as well-mixed populations^18,19^, voluntary participation^18,20,21^, spatial populations^10,22,23^, and network populations^23–25^. A PGG involves participants deciding how many tokens to allocate to a shared pool, in the understanding that all players will be later awarded an equal portion of the public good, regardless of their contributions to the token pool. Hence, the rational, selfish approach is to withhold the tokens and free ride on others’ efforts, but if everyone does this, no one contributes and the public good collapses^26^.

To mitigate this, societies often adopt incentive systems to enforce cooperation within a population. Reward and punishment were traditionally conceptualized as parameters of the outcome structure, with reward reinforcing cooperation by increasing the outcomes from it and punishment penalizing mutual noncooperation by decreasing the outcomes^27,28^.

Hundreds of published studies have been conducted analyzing these strategies under social dilemmas^11,19,29–37^. The literature reviewed reveals two institutional arrangements for incentive strategies^38^: a decentralized system (peer-punishment)^29,39^, in which each player monitors and sanctions/rewards defectors, and a centralized system (pool-punishment), in which the power to administer the incentives is concentrated in a restricted punishment institution^38,40–44^.

Pivotal papers by Fehr and Gächter^29,39^ demonstrated that the introduction of peer-punishment options significantly enhances contributions to public goods, while their removal results in notable declines. However, despite the effectiveness of peer-to-peer punishment and rewards in supporting public goods, their implementation incurs substantial costs, including time, energy, potential danger, and the risk of retaliation^45–47^.

Consequently, while such sanctions benefit the entire cooperating population, the individuals who execute the punishment incur a reduced payoff due to the associated costs. This dynamic allows non-punishing cooperators to effectively benefit from the efforts of punishing cooperators without incurring any cost, a phenomenon termed “second-order free-riding”^33,41,48–50^-in contrast to “first-order free-riding” which is characterized by non-cooperation^43,51^. Therefore, a second-order free-riders punishment (or reward) seems to be the trivial option to sustain a cooperative punishment, but this argument appears vulnerable to infinite regress (e.g., “third-order punishment” to maintain second-order punishment, etc.)^47,48^. In addition, several works mention people appear to be naturally reluctant to punish non-punishers^52–55^. Consequently, some researchers have focused on the pool punishment system considering the vulnerability of peer punishment^41,43^.

In this vein, Sigmund *et al.*^41^ mathematically showed that pool punishment is more stable than peer punishment only when it punishes both first- and second-order free riders, who do not contribute to the punishment system. Additionally, in lab experiments, Traulsen *et al.*^50^ found that participants prefer pool punishment over peer punishment. Moreover, an additional advantage of collecting pool-punishment ahead of the single-order contributions is the fact that second-order free-riders are easily identified, and therefore have a larger propensity to embrace the punishment. In situations where everyone contributes to the public good, second-order free-riders become indistinguishable from peer punishers^43^.

However, the efficacy of these institutions deeply hinges on the support of their members, and theoretical analyses suggest that the free rider problem can be effectively addressed only if individuals who refrain from contributing to the institution are also subject to repercussions^41,42,56^.

Recently, various researchers have investigated other innovative methods to impose penalties on free riders, drawing inspiration from real-world scenarios. Dercole *et al.*^34^ examined a model where defectors are fined a predetermined amount. Sasaki *et al.*^19^ explored the prevention of second-order free riding through the implementation of an entry fee. Chen *et al.*^57^ described a system where a subset of cooperators is randomly chosen to act as punishers. Zhang *et al.*^58^ proposed a mechanism where the financial burden of punishment is distributed among cooperators, and Zhang *et al.*^35^ introduced a maximum limit on the earnings of defectors to encourage cooperative behavior. Likewise, “solitary punisher” punishment increased contributions, according to O’Gorman *et al.*^59^ compared to the baseline scenario. Andreoni and Gee^38^ found that group members often fund a “hired gun” who can sanction the lowest contributor each round. Alventosa *et al.*^60^ examined various centralized institutions under differing payoff schemes and discovered that offering the punisher a contingent payoff leads to a decrease in contributions. Interestingly, this reduction occurs although punishers tend to punish more under such a payoff arrangement. Wang *et al.*^61–63^ have recently explored strategies to minimize the costs associated with sanctioning by optimizing incentives where decentralized systems are in place. Similarly, Xiao *et al.*^64^ have proposed a cost-reduction approach by monitoring a subset of the group rather than the entire population, presenting an efficient method for implementing oversight and sanctioning mechanisms. Although current research has shed light on the effectiveness of centralized incentive systems in promoting cooperation within social dilemmas, several key questions are still open. Among these is the intrinsic relationship between graduated punishment and the monitoring of public goods games.

In her influential work, Nobel laureate Elinor Ostrom^7^ identified that societies effectively addressing collective action problems often implement graduated sanctions, a punitive system designed to intensify based on the severity of the damage inflicted by non-cooperative behavior to communal welfare. Ostrom also pointed to the crucial role of monitoring common-pool resources (CPR), emphasizing that those responsible for overseeing CPR conditions and user conduct must be answerable to the community or act as its representatives. Despite Ostrom’s assertion of these principles as essential for the successful management of shared resources, research exploring these specific strategies remains limited.

Many studies have examined the role of monitoring in punishment systems, yet often they do not fully address its crucial importance, especially given that punishment typically falls upon those detected^65^. Any punitive framework needs to explicitly take into account the available level of oversight system and how it interacts with the severity of the penalties applied.

A recurring assumption in the literature is the existence of a flawless monitoring system, providing complete information and thereby informing punitive decisions accurately. Although reality deviates from this ideal, nowadays, in the “big brother and big data” era of pandemic surveillance, an almost perfect monitoring system is within the realm of possibility.

Moreover, the efficacy of a punishment system is significantly compromised by imprecise monitoring^66^. Yet, an all-seeing monitoring system entails characteristics that, to our understanding, have not been completely leveraged in prior research monitoring systems within a PGG. This could be conceptualized as a “panopticon”, a term devised by Jeremy Bentham^67^, describing a design that enables omniscient observation. Such a system fosters a sense of constant surveillance, thereby strengthening adherence to rules. Implementing a “panopticon” approach to penalize free riders suggests that the mere possibility of punishment serves as a potent deterrent and fosters cooperative behavior.

A powerful unexploited feature of Bentham’s Panoptical is that its architecture not only improves the punishment but also prevents it from using disciplinary actions. The declaration of potential penalties and their actual execution to some defectors serve as a preventative measure. Hence, the penalties suffered by wrongdoers act as vivid reminders to all members of the community of the possible repercussions if they were to commit similar transgressions^68^.

To the best of our understanding, the use of prevention and disciplinary actions, in any form, as a strategy to incentivize cooperation in public goods games has not been extensively studied. However, it undoubtedly constitutes a key element in the toolbox of incentives available to improve cooperation in real-world PGG. A Panoptical implicitly implies a patchwork of cooperation incentives that incorporates a subtle mixture of punishment and discipline^69^.

At this point, it is essential to highlight the slight yet profound divergence between punishment and discipline. Michel Foucault differentiates between punishment and discipline in his analysis of prisons and societal control mechanisms. Punishment refers to the traditional and often public infliction of pain on the body of the convicted individual, which was primarily focused on retribution and deterring others through fear. Discipline, however, operates through systems and procedures that aim to regulate the behavior of individuals, often preemptively, through constant observation and the normalization of behavior. The “panopticon”, thus, is the epitome of disciplinary power, allowing constant observation and the internalization of norms, so that *“... is not is not simply a hinge, a point of exchange between a mechanism of power and a function; it is a way of making power relations functions in a function, and of making a function through these power relations”*^69^.

In essence, while punishment is about responding to transgressions after they occur, discipline is about preventing transgressions before they happen by shaping and controlling behavior to conform to social norms. According to Michel Foucault’s analyses, particularly in “Discipline and Punish”^69^, effective control over a population requires a combination of discipline and punishment, integrated into a larger strategy of power and governance. Both action comprises two indivisible forces of control, converging to a dual force that is not unidirectional but rather exists in a state of constant exchange and interplay, shaping and being shaped by each other in a dynamic equilibrium^70^. Hereinafter, we delineate the term ‘punishment’ as the harsh sanctioning of all wrongdoers, whereas ‘discipline’ refers to the specific deterrence of defection through the penalties experienced by others. These penalties serve as stark reminders to the entire community, illustrating the consequences of wrongful actions.

For Foucault, surveillance and discipline are not merely methods of maintaining order but are central to the exercise of power within societies. In his analysis, the act of surveillance is intertwined with the mechanisms of discipline; it is through being watched that individuals are compelled to conform to societal norms. The constant possibility of observation ensures that individuals internalize these norms, effectively disciplining themselves. Foucault’s seminal work, –*“Surveiller et punir”,* translated into English as “Discipline and Punish”, might also have been aptly rendered as “Monitor and Punish,” reflecting the pervasive theme of surveillance throughout the text. The title underscores the transition from a society where punishment is meted out through public spectacle to one where discipline is enforced through the subtle yet omnipresent gaze of societal institutions. This evolution from overt punishment to covert surveillance-based discipline reveals a profound shift in how power is exerted over individuals, not only penalizing them for transgressions but also shaping their behavior to prevent infractions before they occur^69^.

With the subtitle of the same book *“The birth of the prison”*, Foucault evokes the invention of the main disciplinary technology in humankind’s history —the prison— and with it the birth of a new kind of human organization, the disciplinary society.

Thus, according to Foucault, the prison is concerned with whatever is observable: not only does it wish to display the crime and the criminal but in itself it constitutes surveillance, it is a system of light before being a figure of stone and is defined by *“Panopticism:”* by a visual assemblage and a luminous environment (a central tower surrounded by cells) in which the warder can see all the detainees^70^.

Hence, the prisoner himself begins to be complicit in the control of his behavior due not to what is happening in his environment but to a belief in his own. His belief, in effect, keeps him subjugated. This is panopticism. Realizing what is happening here means realizing the truth of what Foucault said: *“On the loose fibers of the brain lies the unbreakable foundation of the most solid empires”*^69^, indicating that Panoptical surveillance is the final way of punishing. Also, its function is not merely negative, because in addition to preventing infringements, it enables the moral evolution of society. Recently, the importance of such research has also been extended to moral behavior beyond cooperation and has been highlighted by Cappraro and Perc^71^ by arguing that the answer to the question of whether personal norms emerge automatically or require deliberation may not be universal, but rather contingent on the particular behavioral context.

Incorporating these dimensions into the study of cooperation is not only essential but also relatively feasible. Hence, to examine the effects of viewing the Public Good Game as a form of disciplinary technology —essentially, a panoptic prison— we must conceptualize the Public Good Game as both compulsory and perfectly monitored. Let us agree that the most crucial public goods games, such as climate change and pandemics, are ultimately prisons from which we can not escape.

In this context, in our prior study^72^, we have shown that imposing penalties on just a portion of the defectors in a compulsory PGG can induce a reevaluation of behavior among other potential free riders. This approach has been verified as effective for maintaining cooperation within a well-mixed population governed by replicator dynamics, under a framework of centralized punishment. By strategically determining “how many” individuals face penalties, this method not only curtails enforcement expenses but also inadvertently enhances overall cooperation. The main driver behind this phenomenon is primarily attributed to the “panopticon” effect, which involves the implementation of punishment and discipline at a specific trade-off level.

However, the “how many” strategy has a limited ability to regulate the equilibrium between punishment and discipline while it adjusts to a range of population states. Michel Foucault theoretically argues that punishment evolves into discipline as it shifts from being a means of retributive justice, mainly focused on severe retaliation, to a methodical strategy aimed at controlling behavior and promoting conformity among individuals, that aims to shape and control the behavior of individuals in society^70^.

Finally, this paper takes an essential step forward by bringing an important component to this framework: the issue of “how much” defectors should be penalized. We include a variable penalty scale spanning from tiny fines to significant penalties to analyze the balance of punishment and discipline and their effectiveness under different levels of population cooperation.

To the best of our knowledge, this investigation is at the forefront of exploring the intricate interplay between the intensity of penalties (“how much”) and the scope of individuals penalized (“how many”). It offers a more sophisticated perspective through the innovative inclusion of both Foucauldian control strategies aimed at enhancing cooperation in a panoptical well-mixed PGG by replicator dynamics under a single-order centralized punishment system.

## Methods

This paper implements an innovative approach to navigating the intricate web of discipline and punishment mechanisms by implementing a compulsory and fully observable public goods game model integrated with replicator dynamics. We consider it a compulsory observable public goods game, a variation of the classic public goods game in which players are forced to participate while having the ability to observe the behavior or strategy of other players in the game.

Thus, the present model enables an exploration of the complex interplay between the variables of quantity and magnitude within the sanctions framework, thereby providing novel perspectives on the dynamics of cooperative behavior. By concurrently analyzing the scope and intensity of sanctions, we uncover novel avenues for comprehending the equilibrium between these variables in compulsory public goods games, thereby providing valuable academic and pragmatic viewpoints.

Consequently, we examine a well-mixed population in which a public goods game is periodically made available to a random sample of *n* individuals ($n=n_c+n_d$; $n \ge 2$). Each player must determine whether or not to contribute $c=1$ to the game’s common pool. Those who contribute are categorized cooperators ($n_c$), whereas those who do not are categorized defectors ($n_d$). The total contribution is then multiplied by a factor *r* $(1 < r < n)$ and distributed equally among all participants, regardless of whether they contributed.

In a group of $n_c$ cooperators and $n_d$ defectors, the payoffs are as follows^20,30^:

1 $$\begin{aligned} p_c=r\dfrac{n_c}{n}-1, \quad p_d=r\dfrac{n_c}{n}. \end{aligned}$$

The present study focuses on punishing a subset *d* ($0 \le d \le 1$) of defectors. Assuming a random selection of *d* defectors, we propose imposing sanctions on this restricted group while the $(1-d)$ remaining free riders will obtain the normal payoff. This *d* fraction of defectors will have their payoff reduced by *u* ($0 \le u$). The parameter *u* represents the percentage by which the payoff is reduced (e.g., if $u=0.5$, defectors will get half their normal payoff). The penalized defector payoff becomes zero when $u=1$ is established. This condition restores the fractional punishment mechanism as suggested in^72^. Whereas, $d=0$, the game is set as a public goods game with no incentives, as described by^18^.

A defector, unsure whether she will be punished, will have an expected payoff, where with probability $(1-d$) she will have the normal defector payoff, and with probability *d*, her payoff will be reduced by *u*:

2 $$\begin{aligned} p_d= (1-d)r\dfrac{n_c}{n} + d(1-u)r\dfrac{n_c}{n}. \end{aligned}$$

To model the evolution of the strategies, we use the replicator dynamics^73^. Let $0 \le x(t), y(t) \le 1$ be the frequency of each of the corresponding available strategies of the population (cooperators *x* and defectors *y*) at a specific time *t*. To simplify the notation, we drop henceforth the time dependency *t* and simply write *x* and *y*. The frequency distribution of the whole population at a specific time *t* is defined by the state [*x*, *y*] which belongs to the simplex ${{\mathscr {S}}_2}$:

3 $$\begin{aligned} {{\mathscr {S}}_2} = \left\{ [x,y] \in {\mathbb {R}}^2: x,y \ge 0, x+y=1 \right\} . \end{aligned}$$

The interior of ${{\mathscr {S}}_2}$ is defined as the set of points where both strategies could be present (*i.e.*, $0<x$, and $0<y$). The points $x=1$ and $y=1$ represent a homogeneous population of cooperators and defectors, respectively. The evolution of every strategy is contingent upon the replicator dynamic framework, which considers the discrepancy between individual players’ fitness and the population’s average fitness. This framework assumes the presence of a sufficiently large population in which generations blend continuously into one another. Therefore, the system to be analyzed is:

4 $$\begin{aligned} \left\{ \begin{array}{l} {\dot{x}}=x\left( p_x {-} {\bar{p}}\right) \\ {\dot{y}}=y\left( p_y {-} {\bar{p}}\right) \end{array} \right\} , \end{aligned}$$

where ${\bar{p}}=x p_x + y p_y$ is the population’s average payoff.

Consider that the payoff in (2) is defined for a particular number of cooperators. However, in the game, an individual does not know the strategy selected by the group’s other members. To compute a player’s payoff^18,35^, consider that this group composition depends on the frequencies of all strategies in the population (where *x* and *y* represent the frequency of cooperators and defectors respectively). Therefore, in the sample group of size *n*, the probability that *m* of these $n{-}1$ coplayers will be cooperators and the other $n{-}1{-}m$ defectors is given by

5 $$\begin{aligned} \left( {\begin{array}{c}n {-}1\\ m\end{array}}\right) \;x^{m}\;y^{n{-}1{-}m}. \end{aligned}$$

Then, the expected payoff for a defector in a group of *n* players over all possible numbers of cooperators is:

6 $$\begin{aligned} \begin{aligned} p_y&=(1{-}d)\frac{r}{n}\sum _{m=0}^{n {-} 1} m \left( {\begin{array}{c}n{-}1\\ m\end{array}}\right) \left( x\right) ^{m} \left( y\right) ^{n {-}1{-}m} + d(1{-}u)\frac{r}{n}\sum _{m=0}^{n {-} 1} m \left( {\begin{array}{c}n{-}1\\ m\end{array}}\right) \left( x\right) ^{m} \left( y\right) ^{n {-}1{-}m},\\ p_y&= (1{-}d)\frac{r}{n}(n {-} 1)x +d(1{-}u)\frac{r}{n}(n {-} 1)x. \end{aligned} \end{aligned}$$

A similar analysis can be performed to obtain the expected payoff of a cooperator in the population. To distinguish the contribution of one cooperator from the contribution of the other cooperators in the group, the payoff can be rewritten:

7 $$\begin{aligned} p_x=\left( \dfrac{r(n_x{-}1)}{n} {+} \dfrac{r}{n}\right) {-} 1. \end{aligned}$$

where $n_x{-}1$ (the remaining cooperators in the game) is defined as *m*.

8 $$\begin{aligned} \begin{aligned} p_x=\left( \dfrac{rm}{n} {+} \dfrac{r}{n}\right) {-}1 \end{aligned} \end{aligned}.$$

Therefore, the expected payoff for a cooperator in a group of $n \; (n = 2, ..., n)$ players over all possible numbers of cooperators is

9 $$\begin{aligned} \begin{aligned} p_x&=\sum _{m=0}^{n{-}1} \left( {\begin{array}{c}n {-}1\\ m\end{array}}\right) \left( \left( \dfrac{rm}{n} {+} \dfrac{r}{n}\right) {-}1\right) \left( x\right) ^{m} \left( y\right) ^{n{-}1{-}m},\\ p_x&=\sum _{m=0}^{n{-}1} \left( {\begin{array}{c}n {-}1\\ m\end{array}}\right) \left( \dfrac{rm}{n}\right) \left( x\right) ^{m} \left( y\right) ^{n{-}1{-}m}{+}\sum _{m=0}^{n{-}1} \left( {\begin{array}{c}n {-}1\\ m\end{array}}\right) \left( \dfrac{r}{n}\right) \left( x\right) ^{m} \left( y\right) ^{n{-}1{-}m} {-} \sum _{m=0}^{n{-}1} \left( {\begin{array}{c}n {-}1\\ m\end{array}}\right) \left( x\right) ^{m} \left( y\right) ^{n{-}1{-}m},\\ p_x&=\frac{r}{n}(n{-}1)x{+}\dfrac{r}{n}{-}1 =\frac{r}{n}\left( \left( n-1\right) x+1\right) -1. \end{aligned} \end{aligned}$$

The difference in payoff between both strategies shows the relative benefit (or drawback) of cooperators over defectors. This difference, defined as *g*(*d*, *u*, *x*), is given by

10 $$\begin{aligned} \begin{aligned} p_y-p_x=&\left( 1-\frac{r}{n}\right) -dur\left( 1-\frac{1}{n}\right) x=:g(d,u,x). \end{aligned} \end{aligned}$$

In the next Section, we analyze system (4) equilibrium points using those above $p_x$ and $p_y$ payoffs.

## Analysis

In a compulsory public goods game, given that $x+y=1$ and ${\bar{p}}=x p_x + y p_y$, the system can be analyzed with a single equation:

11 $$\begin{aligned} \begin{aligned} {\dot{x}}&=-x(1-x)g(d,u,x)=-x(1-x)(1- (r/n) - durx (1-1/n)). \end{aligned} \end{aligned}$$

For the sake of simplicity, we define a new variable, $\alpha \, =\, du$, that includes parameters *d* and *u*. The equilibrium points of the system (11) are ${\hat{x}}=1$ (${\hat{y}}=0$), ${\hat{x}}=0$ (${\hat{y}}=1$) and, ${\hat{x}}=(n-r)/(r(n-1)\alpha )$. The stability of the equilibrium points is studied with the following lemma.

### Lemma 1

Let us consider Eq. (11) where *x* denotes the frequency of cooperators, *y* the frequency of defectors, $x \ge 0$, $y\ge 0$, respectively; and $x+y=1$. Denoting the fraction of sanctioned defectors by *d*, with $0\le d \le 1$, and the fraction deducted from their payoff by $0\le u$, and defining $\alpha _1 :=(n-r)/(r(n-1))$ then: 1) For $0\le \alpha \le 1$ the equilibrium point ${\hat{x}}=0$ is locally asymptotically stable, 2) ${\hat{x}}=1$ is locally asymptotically stable for $\alpha _1 < \alpha \le 1$ and 3) the point ${\hat{x}}=(n-r)/(r(n-1)\alpha )$, that exists in the interior of the border for $\alpha _1<\alpha$, is an unstable equilibrium point.

### Proof

Consider the Jacobian of the Eq. (11), defined by $J(x,\alpha ) = d{\dot{x}}/dx$, which has the form:

12 $$\begin{aligned} \begin{aligned} J(x,\alpha )&=-(1-2x)(1- (r/n) - \alpha rx (1-1/n))-x(1-x)\alpha r\left( 1-1/n \right) . \end{aligned} \end{aligned}$$

When the expression (12) is evaluated at equilibrium point ${\hat{x}}=0$, it takes the form $J(0,\alpha ) = - (1-r/n)$. Since a game requires $r<n$, the eigenvalue is negative, and the equilibrium point is locally asymptotically stable. This also means that the reciprocal equilibrium point ${\hat{y}}=1$ is locally asymptotically stable since $x+y=1$.

When the Jacobian is evaluated at equilibrium point ${\hat{x}}=1$, the eigenvalue sign depends on the value of $\alpha$, *i.e.*, $J(1,\alpha ) = (1 - r/n) - \alpha r (1-1/n)$. The eigenvalue is negative for $\alpha > \alpha _1$, and therefore ${\hat{x}}=1$ is locally asymptotically stable; however, if $\alpha <\alpha _1$, then the eigenvalue is positive and ${\hat{x}}=1$ is locally unstable.

For simplicity, let denote ${\hat{x}}= (n - r)/(r(n-1)\alpha )$ as ${\tilde{x}}$; then, evaluating the expression (12) at ${\tilde{x}}$:

13 $$\begin{aligned} J({\tilde{x}},\alpha ) = -\left( ((n-r)/n) (n-r-r\alpha (n-1))/r\alpha (n-1)\right) . \end{aligned}$$

Since $0<((n-r)/n)$, the eigenvalue of (13) depends on the value of $(n-r-r\alpha (n-1))/r\alpha (n-1)$. Notice that $(n-r-r\alpha (n-1))$ is negative when $\alpha >(n-r)/(r(n-1))$, *i.e. *$, \alpha >\alpha _1$. In this case, the eigenvalue is positive; therefore, the equilibrium point is unstable. On the contrary, $(n-r-r\alpha (n-1))$ is positive when $\alpha <(n-r)/(r(n-1))$, *i.e.*, $\alpha <\alpha _1$, and consequently, the equilibrium point is stable. However, this case is not possible since the maximum value of *x* is 1 corresponding to $\alpha _1=(n - r)/(r(n - 1))$, that is, the minimum value of $\alpha$ for which the equilibrium point exists. $\square$

**Figure 1:**
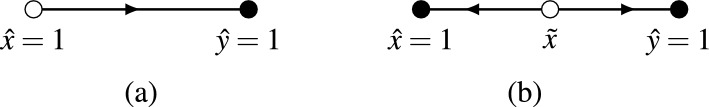
Stability of equilibrium points: black dot represents a stable point, white dot represents an unstable point. (**a**) with $\alpha <\alpha _1$, the ${\hat{x}}=1$ is unstable and ${\hat{y}}=1$ is asymptotically stable. (**b**) if $\alpha _1<\alpha \le 1$, an unstable equilibrium ${\hat{x}}$ (denoted by ${\tilde{x}}$) appears. The value of the equilibrium point is ${\hat{x}}={\tilde{x}}=(n-r)/r(n-1)\alpha$, changing the stability of ${\hat{x}}=1$ that becomes a stable equilibrium point. The possibility of obtaining full cooperation depends on the existence of the interior equilibrium point ${\tilde{x}}$. This equilibrium point appears when $\alpha _1 < \alpha$, and the outcome: full cooperation or full defection will depend on the frequency of cooperators related to the equilibrium ${\tilde{x}}$ (see Fig.  1 b). For values of $\alpha < \alpha _1$, the interior equilibrium point ${\tilde{x}}$ does not exists and consequently the outcome is full defection (see Fig.  1 a).

Figure 1 shows the equilibrium points of the system and their stability. If 0 $\le \alpha < \alpha _1$ then $g(x,\alpha ) \ne 0$ for all values of *x*. In this scenario, there are two equilibrium points, ${\hat{x}}=1$ is globally unstable, and ${\hat{x}}=0$ is globally asymptotically stable (see Fig. 1a). If $\alpha _1 < \alpha$ an unstable equilibrium point ${\tilde{x}}=(n-r)/(r(n-1)\alpha )$ exists (see Fig. 1 b).

As previously mentioned, the function $g(x,\alpha )$ represents the difference between defectors and cooperators’ payoff; then if $p_y>p_x$, the best strategy is to defect, the function $g(x,\alpha )>0$ and the final state of the system is full defection. On the contrary, if $p_x>p_y$, when $g(x,\alpha )<0$; it is better to cooperate, and the system’s final state is full cooperation. As previously established, the function $g(x,\alpha )$ represents the relationship between the initial frequency of cooperators in a group (*x*) and the parameter $\alpha$. To achieve complete cooperation, it is necessary to establish the asymptotic stability of the equilibrium point ${\hat{x}}=1$. To accomplish this, an internal equilibrium point becomes a required condition. The aforementioned point is observed when the value of $\alpha$ is greater than $\alpha _1$.

**Figure 2:**
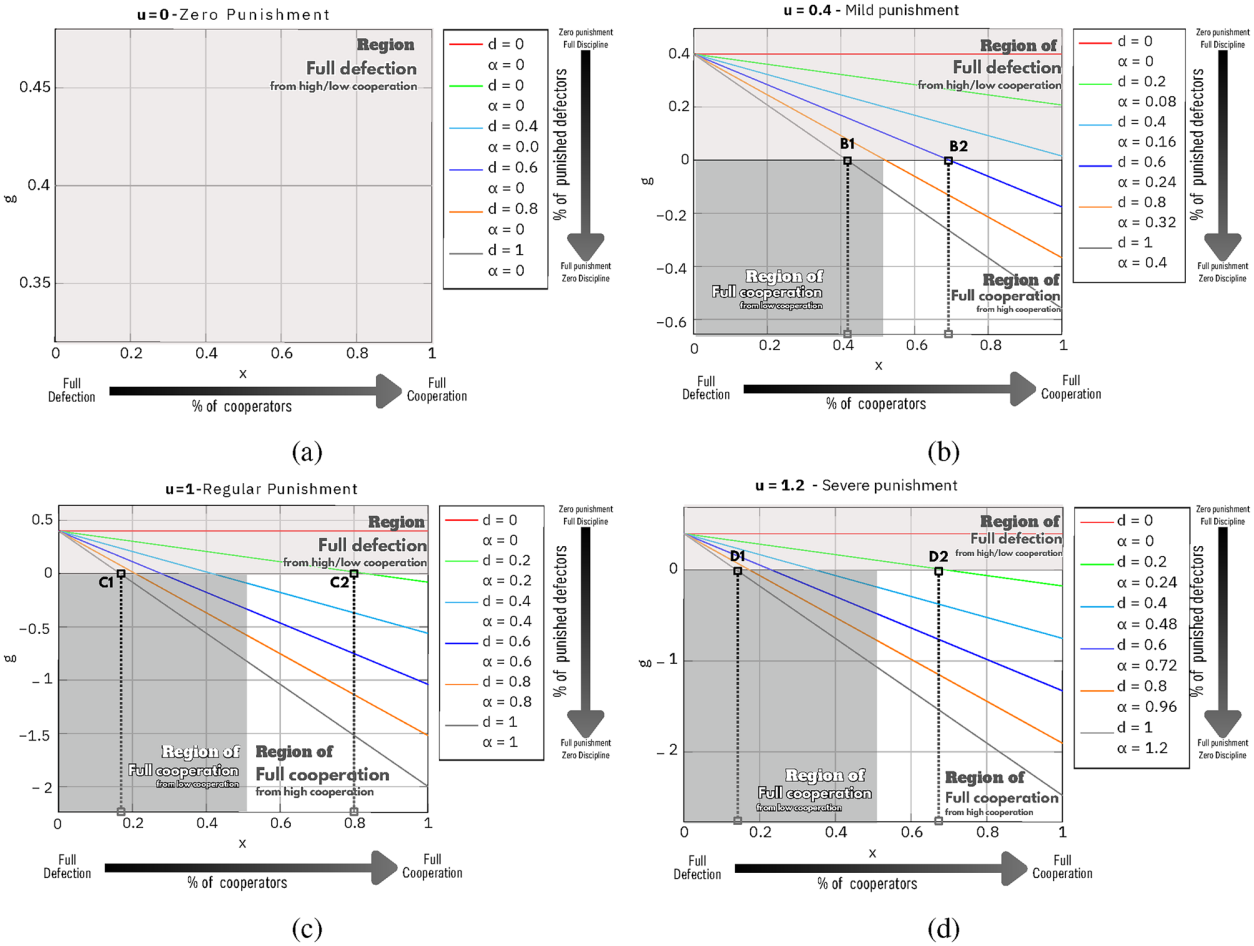
$g(x,\alpha )$ as a function of *x* for a defined value of *u* and several values of *d*: When $g(x,\alpha )>0$, $p_y>p_x$ (defectors prevail), if $g(x,\alpha )<0$, $p_x>p_y$ (cooperators prevail). (**a**) As seen, the population drifts inexorably toward full defection when no defectors are penalized, $g(x,\alpha )>0$ $\forall$ *d*, *x*. (**b**) However, punishment ($d>0$) interacts with *u* and *x* towards full cooperation. In scenario (b) with high defection and *u* = 0.4 (a $40\%$ payoff reduction), full cooperation requires more than $40\%$ baseline and all defectors penalized $(d=1)$ (see point B1). Point B2 indicates that a population with at least $60\%$ sanctioned defectors can reach full cooperation. (**c**) To achieve full cooperation in an uncooperative population, a severe penalty severity ($u=1$) requires initial cooperation of less than $20\%$ and penalizes all defectors (see point C1). A highly cooperative population can achieve $100\%$ cooperation by penalizing $20\%$ of defectors similarly (C2). (**d**) If we impose a 20% a fine cost ($u = 1.2$) on expected payouts for penalized defectors. Only 15% initial cooperation is needed to attain total collaboration in a population unwilling to cooperate under full severe punishment (D1). In a well-behaved population with fractional punishment (20%, $d=0.2$), the initial cooperation level to attain complete cooperation is 70%, a 10% drop from scenario (c) ($x\approx 0.7$ in D2 and $x\approx 0.8$ in C2). Parameters: $r=3$, $n=5$, (**a**) $u=0$, (**b**) $u=0.4$, (**c**) $u=1$, (**d**) $u=1.2$.

The intricate relationship between game parameters *u*, *d*, and *x* in the function $g(x,\alpha )$ is effectively illustrated by Fig. 2. In the scenario where no deduction is made from the defectors’ payoff ($u=0$), as observed in case (a) (see Fig. 2a), the value of *d*, the set of defectors to be punished, is inconsequential, and regardless of the the initial conditions, the population drifts inexorably towards complete defection. This is represented by the constant positive value of the function $g(x,\alpha )$.

Conversely, when punishment is enforced ($u>0$), a critical synergy emerges in the nuanced interplay between *d* and *x*, a vital catalyst for full cooperation. Thus in a population with high defection —as illustrated in scenario (b) (see Fig. 2b)— with *u* set at 0.4 (inflicting a lenient punishment of $40\%$ payoff reduction), we need more than $40\%$ cooperation baseline and all defectors penalized $(d=1)$ to achieve full cooperation (see point B1 in Fig. 2 b). On the other hand, in a population with low defection, with just $60\%$ of defectors sanctioned, full cooperation is feasible (see point B2 in Fig. 2b).

Similarly, scenario (c) (see Fig. 2 c) highlights that in an uncooperative population, a more severe sanction severity ($u=1$) requires an initial cooperation of slightly less than $20\%$ and punishing all the defectors to guarantee full cooperation (refer to point C1 in Fig. 2c). Conversely, complete cooperation can be achieved within a cooperative population by applying the same severity of punishment to merely $20\%$ of the defectors (see point C2 in Fig. 2d).

Lastly, in case (d) (see Fig. 2d), we impose a $20\%$ penalty fee (u = 1.2) over her potential payout paid to the punished defectors. Under these conditions, within a population with great reluctance to cooperate under full punishment, we just need about $15\%$ of initial cooperation and still reach a state of full cooperation (see point D1 in Fig. 2d). Also, it is utmost to note that the mentioned penalty fee, but under a well-behaved population with the same level of fractional punishment ($20\%$ of the population, $d=0.2$), the initial cooperation level to reach full cooperation is reduced to about $70\%$, about $10\%$ compared to the case with a lower punishment severity —scenario (c) ($x\approx 0.7$ in D2 and $x\approx 0.8$ in C2, Fig. 2).

Such evidence affirms that well-tuned punishment can steer populations towards full cooperation, even amidst substantial initial defection. The forthcoming Section will delve into a comprehensive analysis of the implications inherent in the statement above.

## Discussion

The central finding within this paper is that achieving full cooperation does not require universal punishment. The results exposed in the previous Section enlighten this statement by presenting how a well-tuned discipline/punishment scheme can steer populations toward full cooperation, even amidst substantial initial defection by balancing punishment and discipline actions properly.

Nevertheless, the pursuit of cooperation and punishment system design is not confined solely to mathematical models and logical reasoning but also hinges deeply on the philosophical and neurological realms of ethics, power, justice, and sovereignty^74^. Indeed, punishment in the PGG is generally a highly interdisciplinary subject, with significant contributions coming from economics^17,29,38^, social sciences^4,56,75^, network science^24,76,77^, physics^22,36,42,64^, and even moral and ethical studies^71^. Hence, it encompasses a lively interplay between the physical manifestations of actions, the logical aspects that shape their foundation, the moral assessments that assign their worth, and the political structures that sustain and control them^71,78^.

Indeed, the actions of discipline and punishment are political acts that serve as a coercive instrument of authority and the manifestation of power. While it may limit our sovereignty, it simultaneously safeguards our long-term welfare by ensuring that transgressions meet appropriate consequences.

However, as aforementioned, the practical implementation often reveals that not all punishments are uniformly applied or intrinsically fair. An indiscriminate universal sanctioning strategy tends to be resource-intensive and can seldom reach all defectors. After all, no one will be disciplined if we punish everyone. Generalized punishments refer to the harsh, often public, corporal, or capital punishments typical of dysfunctional and fragmented societies. These punishment strategies aimed to inspire dread and discourage behavior by reaching as large a proportion of the wrongdoer population as possible. On the other hand, cohesive and harmonious societies are adopting a more humane approach to punishment that places more emphasis on rehabilitation and change than on punishment per se^69^.

Hence, the punishment/discipline system must be a precision instrument, judiciously applied to engender a sense of equity and equilibrium within the specific population context. Our previously formulated framework introduces a mathematical approach to discipline, where sanctions are tailored rather than uniformly applied, finely tuned to reflect the degrees of defection and the initial state of cooperative behavior. This ensures that punishment functions not as an indiscriminate force but as a considered response that encourages cooperative conduct within a given community. Furthermore, as we have shown in the last section, the consistent perception of potential punishment can significantly alter individual behavior, even in the absence of its uniform application. Hence, our model entails sophisticated and nuanced analysis focusing on the careful tuning of both the extent of punishment and its severity in “observable” Public Goods Games. This public good scenario allows us to mirror the Panoptical idea of Bentham’s surveillance^67^, allowing us to monitor the population. Yet, we deliberately choose to punish only a fraction, thus creating an environment where the uncertainty of punishment becomes a disciplinary force that significant deterrent for defection, and discipline the individuals.

Moreover, Seymour *et al.* highlighted a significant benefit of living within a community: learning the consequences of the punishment by perceiving the impact on the payoff of the sanctioned defectors, even without being punished^74^. Thus, knowledge acquisition via observation holds significant importance, irrespective of whether it pertains to favorable or unfavorable outcomes experienced by others. This understanding may discipline one’s future conduct^79^.

In this context, the questions “how many” and “how much” for the punishment/disciplinary system are central to our discussion. They usher in the broader conversation around justice, proportionality, and efficacy that weaves through the fields of game theory and policy formulation. These considerations are vital in shaping effective strategies to encourage cooperation and manage wrong-doers.

From one perspective, this approach may provoke debates about justice and evoke potential ethical dilemmas, as imposing harsher penalties on fewer individuals could be perceived as inequitable. Notwithstanding, albeit at first glance, it may appear unfair to subject few must suffer punishment to discipline several spotless defectors. However, we must include in the analysis a fundamental virtue that permeates somehow all ethical frameworks−mercy. As a manifestation of compassion and empathy, mercy symbolizes our collective humanity and capacity for forgiveness, and it is among the most widely admired of virtues^80^.

Under the proposed punishment system, by constraining the number of individuals who face sanctions, we inadvertently but simultaneously enhance the exercise of mercy (as well as *d* is the set of penalized defectors, then $(1-d)$ is the set of “redeemed” defectors).

This intricate interplay between dispensing justice and practicing mercy characterizes the multifaceted ethical fabric shaping societal behaviors and norms. Indeed, most influential philosophical views put compassion as the opposite of justice. Therefore, it is critical to comprehend cooperation and its role in group dynamics.

Philosophers such as Hanna Arendt, Jeremy Betham, Friedrich Nietzsche, and Michel Foucault made keynote contributions to power, morality, and punishment. Their works often overlap thematically, although their views on mercy and punishment may diverge^80^.

On the one hand, Nietzsche considered mercy a sign of weakness. Works such as^81^ criticize mercy because it encourages servile morality and a victim mentality. He suggests that shades of unconditional or unilateral forgiveness do not necessarily arise from a desire to live together but are rooted in and shaped by our need to calm ourselves for the inevitable narcissistic damage we endure in our battle for recognition.

In dyadic interactions, Nietzsche presents a contrasting viewpoint by asserting that mercy benefits the bestower rather than the recipient. If the statement is accurate, it is suggested that we should not perceive mercy as an altruistic virtue but rather as a virtue rooted in pride. According to Nietzsche, the concept of “mercy” is considered a virtue among rulers, indicating their ability to endure a tangible experience of loss as evidence of their authority^81^.

In this vein, in^81^, the author emphasizes that mercy involves a shift from relying on recognition from others to fixing the hole in the order of rank that previously protected us from vulnerability and fragility: *“The creditor always becomes more human to the extent that he has grown richer; finally, how much injury he can endure without suffering from it becomes the actual measure of his wealth. It is not unthinkable that a society might attain such a consciousness of power that it could allow itself the noblest luxury possible—letting those who harm it go unpunished. “What are my parasites to me” it might say. “May they live and prosper: I am strong enough for that’... This self-overcoming of justice: one knows the beautiful name it has given itself—mercy; mercy remains the privilege of the most powerful man, or better, his—beyond the law.”*

Arendt somehow expands this argument and adds the necessity of identifying a political framework centered around the concept of mercy by examining the historical practices of pardoning and amnesty^82^. The author emphasizes the importance of the Roman principle of sparing the defeated (*parcere subiectis*) as an introductory hint that forgiveness is a crucial treatment for the unavoidable consequences of human acts. Even the Roman Stoic philosophy does not release trespassers from wrongs but spares the vanquished final destruction; thus, it cannot be considered a sign of forgiveness.

Notwithstanding, Arendt acknowledges this when she adds that we should also see *“the possibility to commute the death sentence,”* which also has Roman roots, as a rudimentary sign of forgiveness. This can also be understood as a system for controlling and adjusting sanctions and, by doing so, increasing the uncertainty of the punishment.

In this context, Bentham believes that pardons tend to increase uncertainty by undermining the confidence of the potential wrongdoer that she will ultimately be punished for her conduct. According to this author, any such uncertainty must be explicitly compensated for by an increase in the severity of the punishment^68^.

This later analysis converges the proposal presented in Botta* et al.*^72^, where fractional punishment emerges as a beneficial mechanism under resource-scarce conditions. Still, if defectors invade the population (Fig. 2c), even under full-scope punishment $(d=1)$, the fulfillment of total cooperation remains unreachable.

Within this framework, the concept of “biopower” coined by Michel Foucault becomes significant^83^. In contrast to punitive power, which is directed towards individuals within a mechanistic framework, biopower aims at entire populations using an organic approach. Biopower, by explicitly considering society (“life”) as a subject of political-economic analysis, caused the actions of history to impact the evolution of moral societies. Foucault referred to this interference as biohistory^84^.

Thus, fractional punishment, which attempts to serve as both punishment and discipline, must take into account the biohistory of the society, in this case, the population, to have a successful influence on the evolution of societies toward cooperation, or modernity, as defined by Foucault.

Then, in this work, we develop a strategy to achieve full cooperation in compulsory PGG that boils down to adjusting both the harshness of the punishment ($u>1$) and the fraction of punished defectors according to the population cooperation level (a Markovian version of the biohistory). The mathematical model simulation results, as depicted in the previous section, appeal to be consistent with the Foucauldian structure of punishment, discipline, biopower, and biohistory.

Finally, the proposed model might contribute to policymakers governing Public Goods Games to adjust the coverage (*d*) and severity (*u*) of punishment dynamically, effectively balancing punishment and discipline, steering the evolution of cooperation under the premise that everyone is under surveillance. 

According to Foucault: *“Justice must always question itself, just as society can exist only employing the work it does on itself and its institutions.”*^85^, Therefore, he implies that justice, like society, is not something fixed but rather something that needs constant introspection. He argues that justice, to be fair and relevant, must constantly evaluate its principles, procedures, and results. Therefore, what is fair and what is not could change, such as the severity of punishment in our model. In this setting, self-questioning and institutional work become an internal discipline that keeps society and its judicial systems dynamic, flexible, and aligned with changing behavior and ethics.

Finally, our work delves into punishment, discipline, justice, power, and cooperation, offering a nuanced understanding of these concepts and a rigorous framework for their application. It raises profound questions about fairness in cooperative scenarios, the use of power in managing cooperation, and designing systems that balance individual and collective interests while promoting cooperation. The philosophical implications of this study offer a fertile ground for further exploration and debate.

## Conclusion

Far from mere instruments of coercion, discipline, and punishment structures are crucial safeguards in our society, nurturing cooperation and upholding justice. The crux of this research is the crucial nature of cooperation in social systems and its inherent difficulties in public good scenarios. Such situations are inherently complex, with voluntary contributions from individuals often offset by the temptation of free-riding. A theoretical cornerstone, the public goods game, encapsulates this dilemma by presenting a sensitive dynamic of individual rational choices that, when aggregated, can precipitate the collective’s demise—the tragedy of the commons. Amidst this, the call to foster cooperation rings loud, urging individuals to shift their focus from immediate personal gain to a more collective, sustained, long-term welfare perspective.

To tackle this, our study brings forward an innovative angle on using Panoptic and compulsory PGG under incentive mechanisms inspired by Foucauldian theories of discipline and biopower to underpin cooperation. In this vein, we delve into the theoretical underpinnings and mathematical constructs underlying punishment and discipline mechanisms.

Hence, in a numerical simulation, we show an approach to tailored-made punishment—well-tuning its reach and severity—that can nudge a population towards full and stable cooperation, even in an initial population with an extensive defection rate. Our discussion unravels the subtleties of this approach and underlines the potential importance of our mathematical model in engineering the Foucauldian biopower, discipline and punishment towards cooperation, justice, power, and mercy in this complex landscape.

The main finding of our model is that achieving full cooperation within a population does not necessitate universal and equitable punishment-a notion often impractical due to scalability issues and associated costs. Instead, a tailored balance between the number of individuals penalized and the intensity of their sanctions under panoptical surveillance is crucial. This approach considers the prevailing level of cooperation within the population, suggesting that strategic, targeted disciplinary actions can more effectively cultivate widespread cooperative behavior.

## Data Availability

All data generated or analyzed during this study are included in this published article.

## References

[1] Kennedy D, Norman C (2005). What don’t we know?. Science.

[2] Hamilton WD (1964). The genetic evolution of social behavior. i and ii. J. Theor. Biol..

[3] Trivers RL (1971). The evolution of reciprocal altruism. Q. Rev. Biol..

[4] Dawes RM (1980). Social dilemmas. Annu. Rev. Psychol..

[5] Hardin G (1968). The tragedy of the commons. Science.

[6] Axelrod R, Hamilton WD (1981). The evolution of cooperation. Science.

[7] Ostrom E (1990). Governing the Commons: The Evolution of Institutions for Collective Action.

[8] Nowak M, Sigmund K (2005). Evolution of indirect reciprocity. Nature.

[9] Perc M, Gómez-Gardenes J, Szolnoki A, Floría LM, Moreno Y (2013). Evolutionary dynamics of group interactions on structured populations: a review. J. R. Soc. Interface.

[10] Nowak MA, May RM (1992). Evolutionary games and spatial chaos. Nature.

[11] Andreoni J, Harbaugh W, Vesterlund L (2003). The carrot or the stick: Rewards, punishments, and cooperation. Am. Econ. Rev..

[12] Von Neumann J, Morgenstern O (1944). Theory of Games and Economic Behavior.

[13] Smith JM (1986). Evolutionary game theory. Physica D.

[14] Taylor PD, Jonker LB (1978). Evolutionary stable strategies and game dynamics. Math. Biosci..

[15] Zeeman, E.C. Population dynamics from game theory. In *Global Theory of Dynamical Systems*, pp. 471–497 (Springer Berlin Heidelberg, 1980).

[16] Weibull JW (1997). Evolutionary Game Theory.

[17] Kagel JH, Roth AE (2020). The Handbook of Experimental Economics.

[18] Hauert C, De Monte S, Hofbauer J, Sigmund K (2002). Volunteering as Red Queen mechanism for cooperation in public goods games. Sci. Newy. NY..

[19] Sasaki T, Brännström A, Dieckmann U, Sigmund K (2012). The take-it-or-leave-it option allows small penalties to overcome social dilemmas. Proc. Natl. Acad. Sci. U.S.A..

[20] Hauert C, De Monte S, Hofbauer J, Sigmund K (2002). Replicator dynamics for optional public good games. J. Theor. Biol..

[21] Szabó G, Hauert C (2002). Evolutionary prisoner’s dilemma games with voluntary participation. Phys. Rev. E.

[22] Perc M (2017). Statistical physics of human cooperation. Phys. Rep..

[23] Nowak MA, Tarnita CE, Antal T (2010). Evolutionary dynamics in structured populations. Philos. Trans. R. Soc. B Biol. Sci..

[24] Szabó G, Fáth G (2007). Evolutionary games on graphs. Phys. Rep..

[25] Santos FC, Pacheco JM (2005). Scale-free networks provide a unifying framework for the emergence of cooperation. Phys. Rev. Lett..

[26] Sigmund K (2007). Punish or perish? Retaliation and collaboration among humans. Trends Ecol. Evol..

[27] Shinada M, Yamagishi T (2007). Punishing free riders: Direct and indirect promotion of cooperation. Evol. Hum. Behav..

[28] Rapoport A (1967). A note on the index of cooperation for prisoner’s dilemma. J. Conflict Resolut..

[29] Fehr E, Gächter S (2000). Cooperation and punishment in public goods experiments. Am. Econ. Rev..

[30] Sigmund K, Haiden N, Hauert C (2004). The dynamics of public goods. Discret. Contin. Dyn. Syst. Series B.

[31] Sigmund K (2010). The Calculus of Selfishness.

[32] Hilbe C, Sigmund K (2010). Incentives and opportunism: From the carrot to the stick. Proceedings. Biol. Sci. R. Soc..

[33] Hilbe C, Traulsen A, Rohl T, Milinski M (2014). Democratic decisions establish stable authorities that overcome the paradox of second-order punishment. Proc. Natl. Acad. Sci..

[34] Dercole F, De Carli M, Della Rossa F, Papadopoulos AV (2013). Overpunishing is not necessary to fix cooperation in voluntary public goods games. J. Theor. Biol..

[35] Zhang J, Zhu Y, Li Q, Chen Z (2018). Promoting cooperation by setting a ceiling payoff for defectors under three-strategy public good games. Int. J. Syst. Sci..

[36] Helbing D, Szolnoki A, Perc M, Szabó G (2010). Punish, but not too hard: How costly punishment spreads in the spatial public goods game. New J. Phys..

[37] Szolnoki A, Perc M (2013). Effectiveness of conditional punishment for the evolution of public cooperation. J. Theor. Biol..

[38] Andreoni J, Gee LK (2012). Gun for hire: Delegated enforcement and peer punishment in public goods provision. J. Public Econ..

[39] Fehr E, Gächter S (2002). Altruistic punishment in humans. Nature.

[40] Brandt H, Hauert C, Sigmund K (2006). Punishing and abstaining for public goods. Proc. Natl. Acad. Sci. U.S.A..

[41] Sigmund K, De Silva H, Traulsen A, Hauert C (2010). Social learning promotes institutions for governing the commons. Nature.

[42] Szolnoki A, Szabo G, Perc M (2011). Phase diagrams for the spatial public goods game with pool punishment. Phys. Rev. E.

[43] Perc M (2011). Sustainable institutionalized punishment requires elimination of second-order free-riders. Sci. Rep..

[44] Wang X, Duh M, Perc M (2020). Pool expulsion and cooperation in the spatial public goods game. Phys. Lett. A.

[45] Janssen MA, Bushman C (2008). Evolution of cooperation and altruistic punishment when retaliation is possible. J. Theor. Biol..

[46] Nelissen RM (2008). The price you pay: Cost-dependent reputation effects of altruistic punishment. Evol. Hum. Behav..

[47] Barclay, P. & Kiyonari, T. Why sanction? functional causes of punishment and reward. *Reward. Punishm. Soc. Dilemmas* pp. 182–196 (2014).

[48] Ozono H, Kamijo Y, Shimizu K (2017). Punishing second-order free riders before first-order free riders: The effect of pool punishment priority on cooperation. Sci. Rep..

[49] Sasaki T, Unemi T (2011). Replicator dynamics in public goods games with reward funds. J. Theor. Biol..

[50] Traulsen A, Rohl T, Milinski M (2012). An economic experiment reveals that humans prefer pool punishment to maintain the commons. Proceed. R. Soc. B Biol. Sci..

[51] Yamagishi T (1986). The provision of a sanctioning system as a public good. J. Pers. Soc. Psychol..

[52] Barclay P (2006). Reputational benefits for altruistic punishment. Evol. Hum. Behav..

[53] Cinyabuguma M, Page T, Putterman L (2006). Can second-order punishment deter perverse punishment?. Exp. Econ..

[54] Kiyonari T, Barclay P (2008). Cooperation in social dilemmas: free riding may be thwarted by second-order reward rather than by punishment. J. Pers. Soc. Psychol..

[55] Nikiforakis N, Normann H-T (2008). A comparative statics analysis of punishment in public-good experiments. Exp. Econ..

[56] Gross J, Méder ZZ, Okamoto-Barth S, Riedl A (2016). Building the leviathan-voluntary centralisation of punishment power sustains cooperation in humans. Sci. Rep..

[57] Chen X, Szolnoki A, Perc M (2014). Probabilistic sharing solves the problem of costly punishment. New J. Phys..

[58] Zhang, J., Zhu, Y., Chen, Z. & Cao, M. Evolutionary Game Dynamics Driven by Setting a Ceiling in Payoffs of Defectors. In *Proceedings of the 36th Chinese Control Conference July 26-28, 2017, Dalian, China*, 11289–11295 (2017).

[59] O’Gorman R, Henrich J, Van Vugt M (2009). Constraining free riding in public goods games: designated solitary punishers can sustain human cooperation. Proceed. R. Soc. B Biol. Sci..

[60] Alventosa A, Antonioni A, Hernández P (2021). Pool punishment in public goods games: How do sanctioners’ incentives affect us?. J. Econ. Behav. Organ..

[61] Wang S, Chen X, Xiao Z, Szolnoki A, Vasconcelos VV (2023). Optimization of institutional incentives for cooperation in structured populations. J. R. Soc. Interface.

[62] Wang S, Chen X, Szolnoki A (2019). Exploring optimal institutional incentives for public cooperation. Commun. Nonlinear Sci. Numer. Simul..

[63] Wang S, Chen X, Xiao Z, Szolnoki A (2022). Decentralized incentives for general well-being in networked public goods game. Appl. Math. Comput..

[64] Xiao J, Liu L, Chen X, Szolnoki A (2023). Evolution of cooperation driven by sampling punishment. Phys. Lett. A.

[65] DeAngelo G, Gee LK (2020). Peers or police?: The effect of choice and type of monitoring in the provision of public goods. Games Econom. Behav..

[66] Grechenig K, Nicklisch A, Thöni C (2010). Punishment despite reasonable doubt-a public goods experiment with sanctions under uncertainty. J. Empir. Leg. Stud..

[67] Bentham, J. *Panopticon Or the Inspection House*. No. v.2 in Panopticon Or the Inspection House (1791).

[68] Bentham J (2009). The Rationale of Punishment.

[69] Foucault, M. Discipline and punish. In *Social Theory Re-Wired*, pp. 291–299 (Routledge, 2023).

[70] Deleuze G (1988). Foucault.

[71] Capraro V, Perc M (2021). Mathematical foundations of moral preferences. J. R. Soc. Interface.

[72] Botta R, Blanco G, Schaerer CE (2021). Fractional punishment of free riders to improve cooperation in optional public good games. Games.

[73] Hofbauer J, Sigmund K (1998). Evolutionary Games and Population Dynamics.

[74] Seymour B, Singer T, Dolan R (2007). The neurobiology of punishment. Nat. Rev. Neurosci..

[75] Ostrom E (2002). Chapter 24 Common-pool resources and institutions: Toward a revised theory. Handb. Agric. Econ..

[76] Duh M, Gosak M, Perc M (2021). Public goods games on random hyperbolic graphs with mixing. Chaos Solitons Fractals.

[77] Gomez-Gardenes, J., Romance, M., Criado, R., Vilone, D. & Sánchez, A. Evolutionary games defined at the network mesoscale: The public goods game. *Chaos: An Interdisciplinary J. Nonlinear Sci.* **21** (2011). 10.1063/1.3535579 21456855

[78] Kumar A, Chowdhary S, Capraro V, Perc M (2021). Evolution of honesty in higher-order social networks. Phys. Rev. E.

[79] Vasconcelos VV, Dannenberg A, Levin SA (2022). Punishment institutions selected and sustained through voting and learning. Nat. Sustain..

[80] Tuckness A, Parrish JM (2014). The Decline of Mercy in Public Life.

[81] Nietzsche F (1998). On the genealogy of morality.

[82] Arendt H (1958). The Human Condition.

[83] Foucault, M. *Security, Territory, Population: Lectures at the Collège de France, 1977-78* (Springer, 2007).

[84] Lawlor L, Nale J (2014). The Cambridge Foucault Lexicon.

[85] Foucault, M. Vous êtes dangereux. *Liberation* **639** (1983).

